# Giant Duodenal Metastatic Gastrointestinal Stromal Tumor (mGIST) in a Young Man

**DOI:** 10.7759/cureus.71162

**Published:** 2024-10-09

**Authors:** Stanko J Baćo, Igor A Stakic, Jovica Mišić, Sonja Đukanović

**Affiliations:** 1 General Surgery, Public Health Institution Hospital “Dr Mladen Stojanovic”, Prijedor, BIH; 2 Surgery, Private Hospital, Banja Luka, BIH; 3 General Surgery, Saint Luke the Apostle Hospital, Doboj, BIH; 4 Emergency, Public Health Institution Dom Zdravlja Prijedor, Prijedor, BIH

**Keywords:** duodenal gastrointestinal stromal tumors, gastrointestinal stromal tumor (gist), giant abdominal tumor, unresectable tumors, young men tumor

## Abstract

Gastrointestinal stromal tumors (GISTs) are rare neoplasms affecting the gastrointestinal (GI) tract, primarily in middle-aged and elderly individuals. Originating from interstitial cells of Cajal (ICC), they can develop anywhere in the GI tract, with varying symptoms. Diagnosis involves imaging and tissue acquisition, with surgical excision and tyrosine kinase inhibitors as treatment options. Recurrence rates are high, particularly in larger tumors, which have a significant impact on survival rates. Neoadjuvant therapy may be beneficial in advanced cases. The recent World Health Organization (WHO) classification categorized all GISTs as malignant, emphasizing the importance of prompt diagnosis and management. We are presenting a case of a 24-year-old male who presented with abdominal discomfort and a sensation of pressure while bending forward. The abdominal mass, which was equivalent to the size of a basketball, was disclosed by the physical and radiological examinations. This mass pressurized the surrounding organs and filled the abdominal cavity. During the surgery, we found a well-defined, clearly localized, irregular spherical tumor with two metastases in the greater omentum. The tumor had numerous diverticular expansions of varying diameters. Despite the tumor's substantial size and proximity to adjacent structures and organs, we were able to successfully excise the tumor entirely (R0) and interruptedly suture the duodenum after a partial D4 resection, the tumor's point of origin. The postoperative period was unremarkable. We discharged the patient without any complications and commenced imatinib therapy. During the seven months following the operation, the patient was on adjuvant imatinib therapy and maintained recurrence-free.

## Introduction

Gastrointestinal stromal tumors (GISTs) are non-epithelial neoplasms that affect the gastrointestinal (GI) tract. These mesenchymal tumors represent around 1% of all primary malignant tumors of the gastrointestinal tract, although they represent the most prevalent form of GI mesenchymal tumors. They are most common among the middle-aged and elderly (median age 60-65 years), with a little male predominance (3:1) [1]. Tumor cells develop from the spindle-shaped mesenchymal cells called interstitial cells of Cajal (ICCs) or stem cell precursors to these cells. ICCs are located within the gastrointestinal tract wall and the enteric division of the autonomic nervous system [2,3]. These cells, also referred to as gastrointestinal pacemaker cells, basically regulate peristaltics [1,4]. The incidence varies according to the geographical area. In Europe, the annual incidence of newly diagnosed GISTs is typically seven to 15 cases per million people [4]. Even though GIST can arise anywhere, the stomach and small intestine are the most frequent locations. In addition to originating from the digestive tract (colon, rectum, and appendix combined - 5%, and esophagus 2% to 3%), they may also derive from other organs (mesentery, omentum, retroperitoneum, and pancreas), where stem cells differentiate into ICCs [2,3]. The location and size of the tumor determine its clinical symptoms. Small-size tumors are usually asymptomatic and discovered by accident during an endoscopic examination, on radiological imaging, or during surgery [2]. Nonspecific symptoms include early satiety, anemia, abdominal discomfort, and upper gastrointestinal hemorrhage/hemoperitoneum. Cases of GISTs are mostly sporadic and about 5% are part of family genetic syndromes [2]. Depending on the tumor size, tumor location, and mitotic index, GISTs are categorized as low risk or high risk rather than being classified as benign or malignant. Their growth can be either slow and indolent or quick, metastasizing, and potentially fatal. The prognosis may be favorable if diagnosed and treated promptly. The recent 2020 World Health Organization (WHO) Classification categorizes all GISTs as malignant, irrespective of their size, origin, or mitotic index [4].

The diagnosis is made based on imaging and endoscopic examinations, and tissue acquisition using immunohistochemistry staining confirms the diagnosis. Surgical excision is the preferred therapeutic option. Tyrosine kinase inhibitors are also available, and they are particularly effective in treating big, unresectable, and metastatic GISTs [4,5].

## Case presentation

We present the case of a 24-year-old young man who presented to our surgical ambulance with an indeterminate sensation of abdominal discomfort. The patient's main symptom was an unpleasant feeling of pressure when leaning or bending forward. After months, the symptoms intensified in the week prior to the evaluation. No additional complaints had been expressed. The patient was in perfect health, without changes in bowel habits, did not take any daily medications, and had never undergone surgery. There was no familial history of malignant disease. The physical examination revealed a basketball-sized, painless abdominal tumor. All vital signs were stable. Renal and liver function tests, cancer antigen 19-9 (CA-19.9), carcinoembryonic antigen (CEA), and serum amylase were normal.

**Figure 1:**
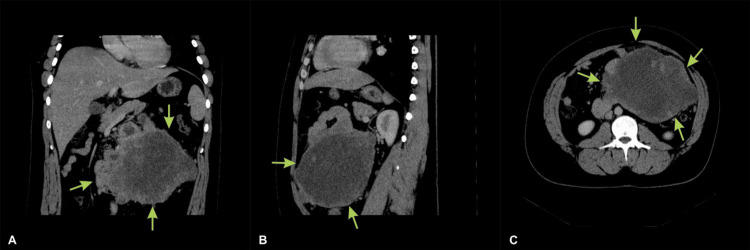
Contrast-enhanced computed tomography (CECT) of the abdomen and pelvis: (A) coronal view, (B) sagittal view, and (C) axial view showing an irregular tumor occupying the abdominal cavity (yellow arrows) and in close touch with the surrounding structures and the abdominal wall.

Abdominal and pelvis contrast-enhanced computed tomography (CECT) revealed an abdominal mass measuring approximately 25x20 cm that was suggestive of GIST. The tumor mass occupied the entire abdominal cavity and compressed the abdominal organs, particularly the small and large intestines (Figures 1A-1C). The mass invaded the small intestine, specifically the D4 part of the duodenum.

**Figure 2:**
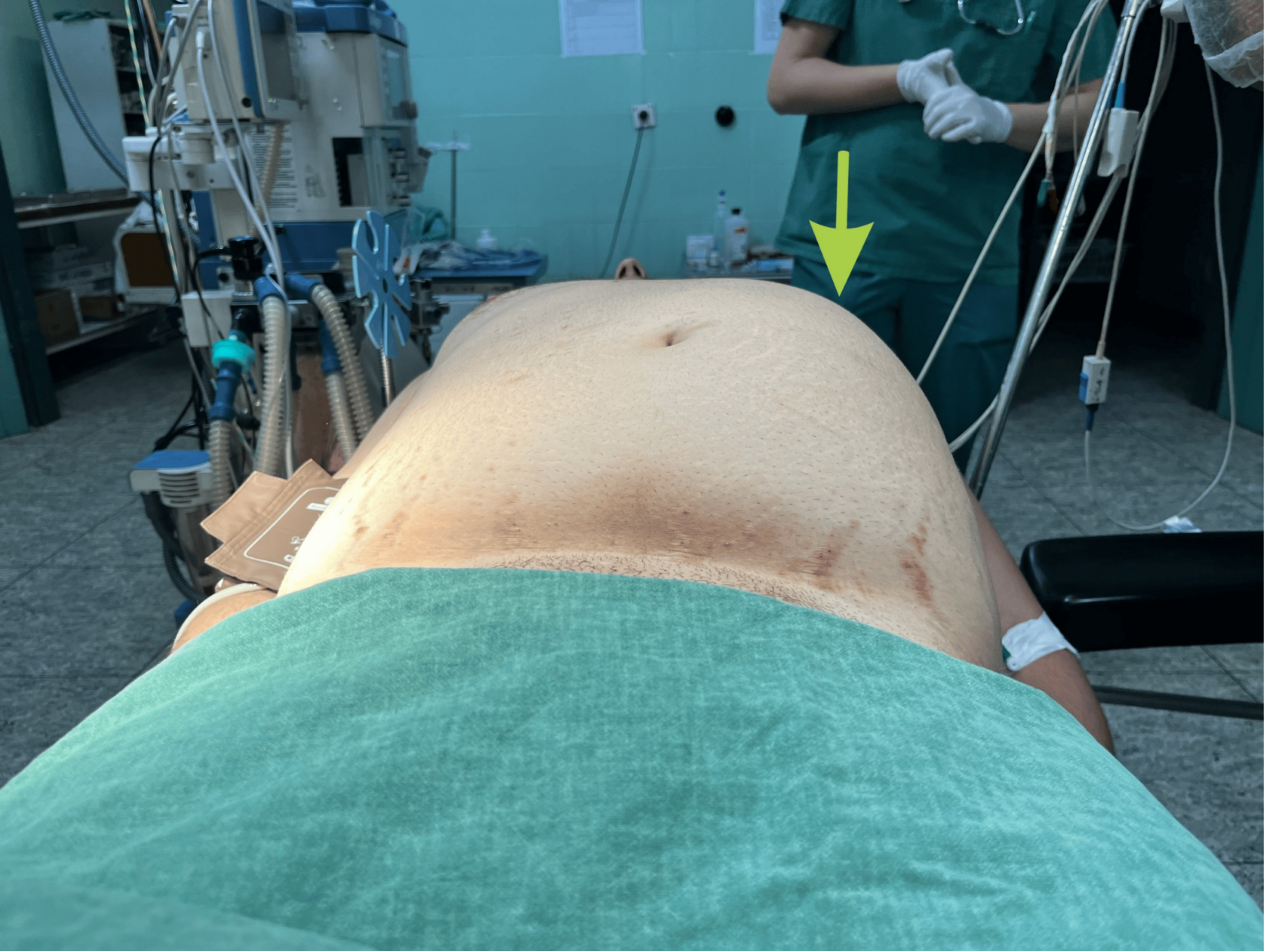
Preoperatively observed distension of the left hemiabdomen (green arrow)

We transported the patient to the explorative laparotomy. Preoperatively, the distension of the left hemiabdomen could be seen (Figure 2).

**Figure 3:**
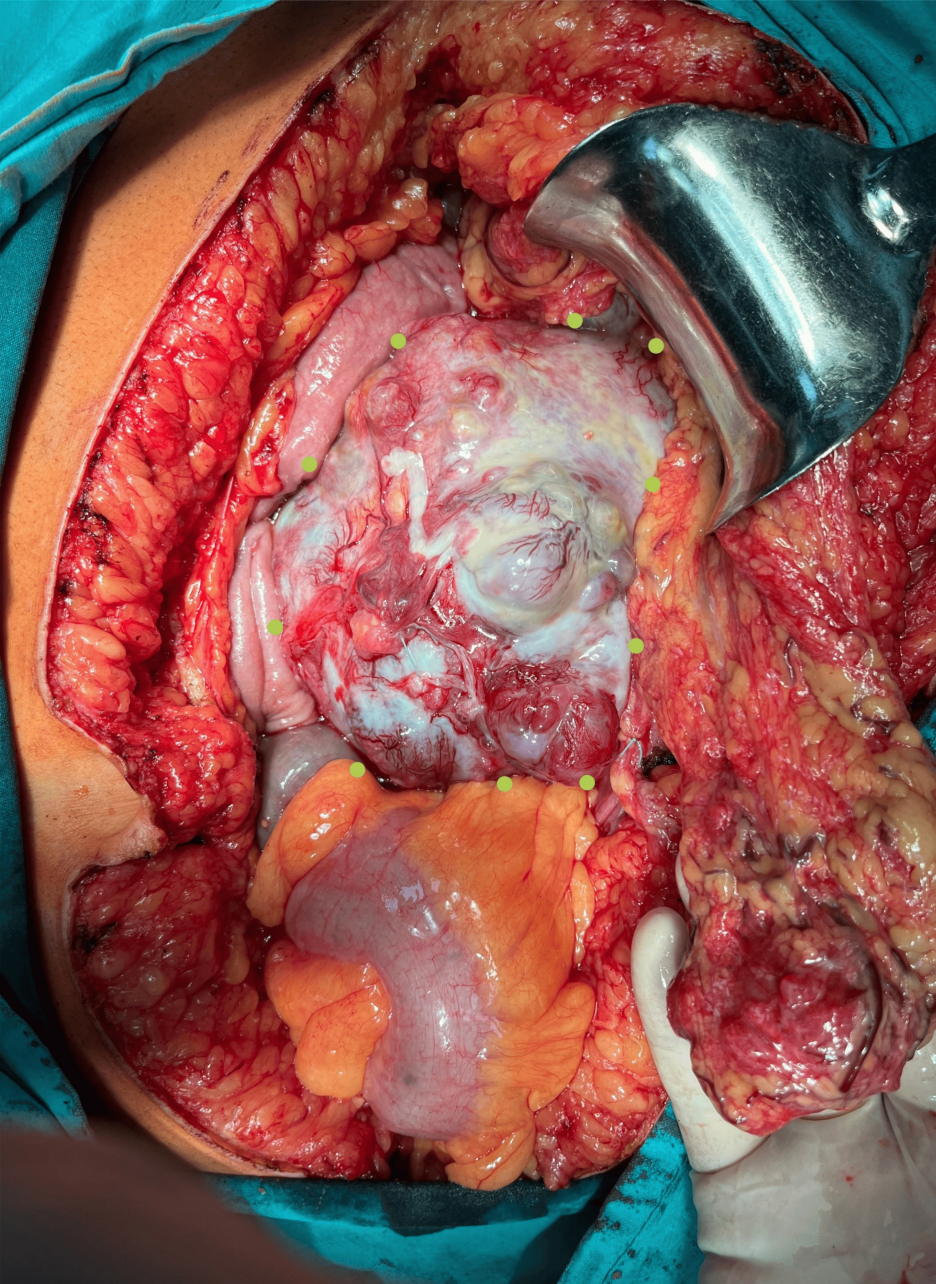
Intraoperative image of the tumor (green dots indicate the borders of the tumor).

**Figure 4:**
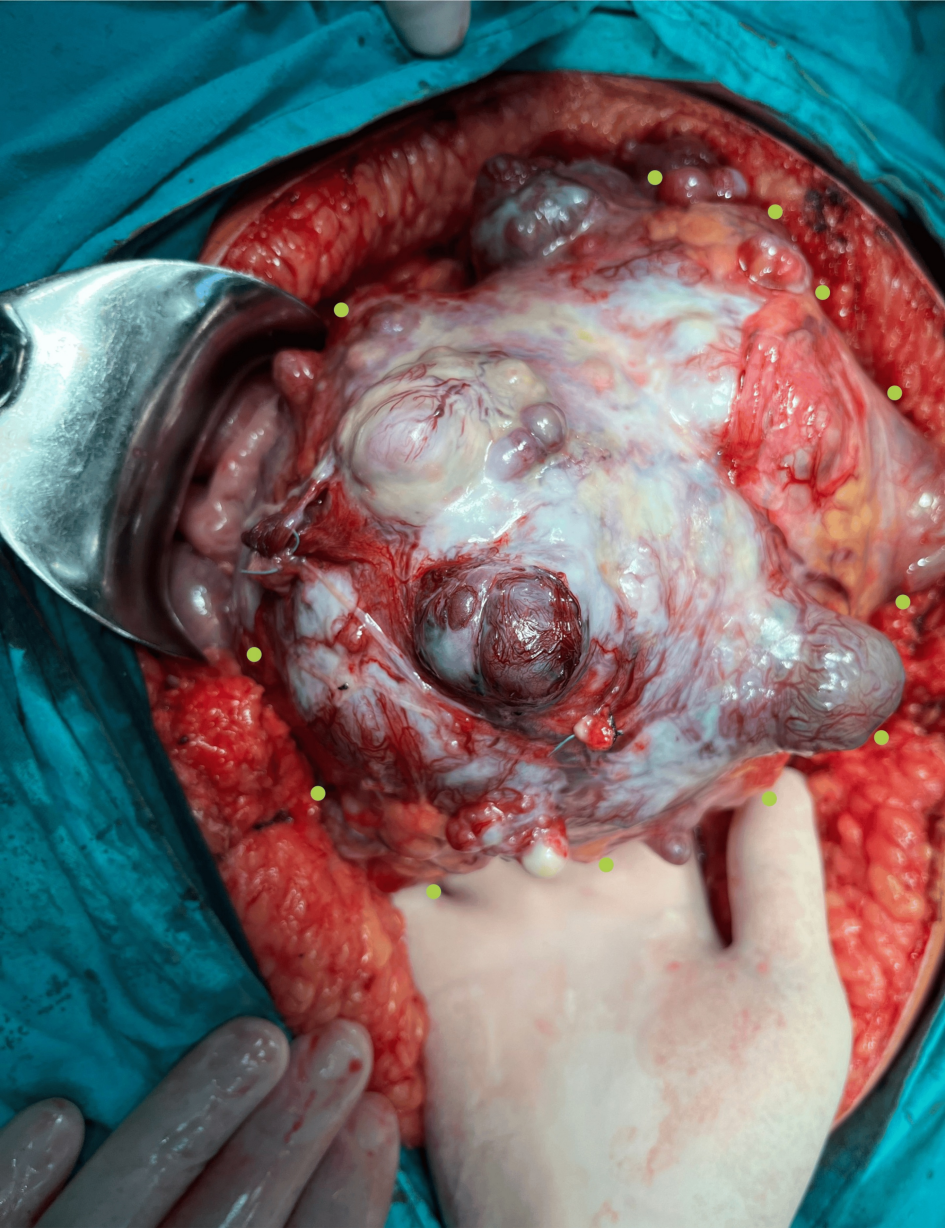
An irregular spherical tumor with numerous diverticular expansions of varying sizes (green dots indicate the borders of the tumor).

**Figure 5:**
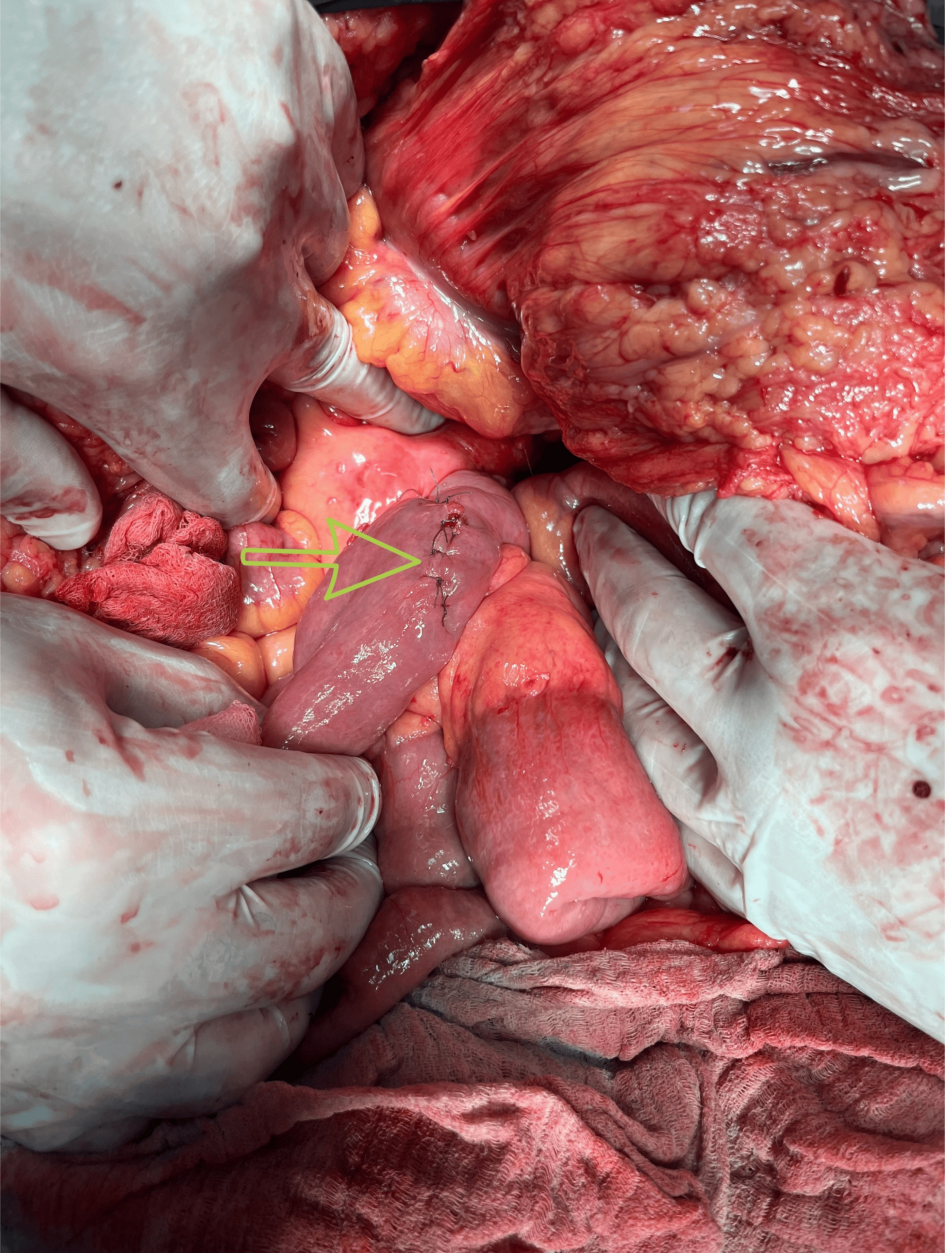
Sutured duodenum D4 after a partial resection (green arrow)

**Figure 6:**
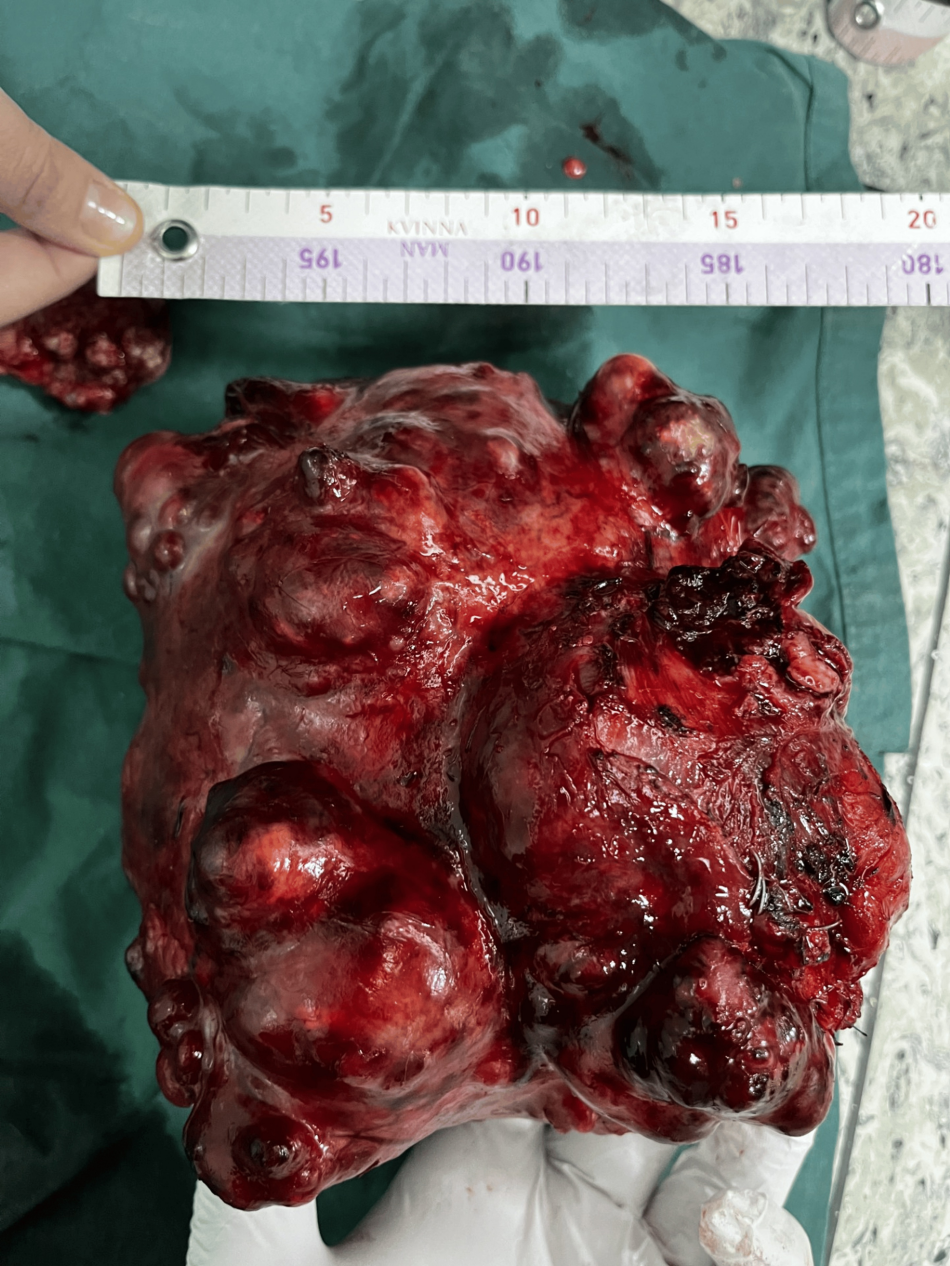
Specimen: an irregular spherical tumor with numerous diverticular expansions of varying sizes, 20 cm in width

**Figure 7:**
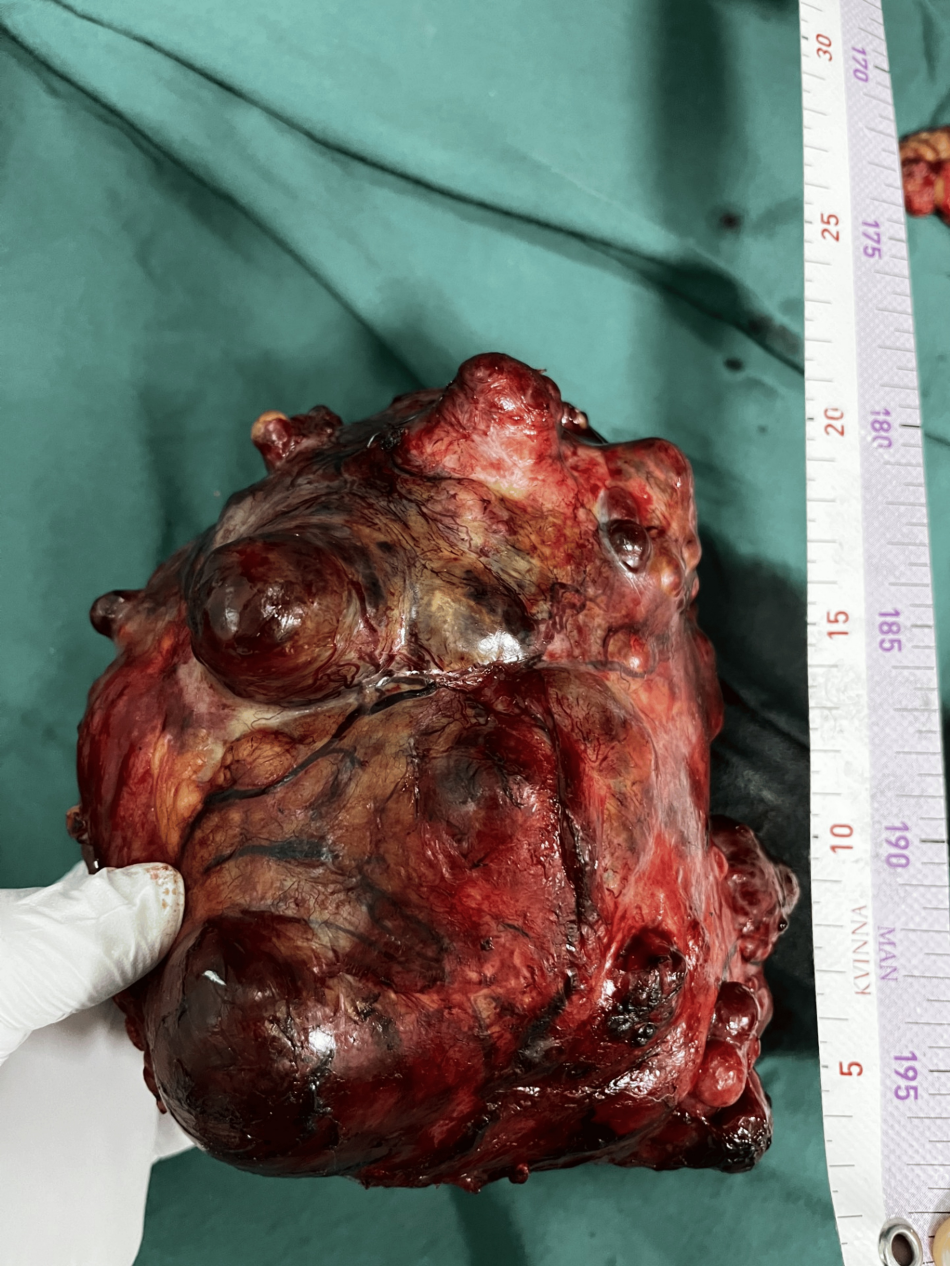
Specimen: an irregular spherical tumor with numerous diverticular expansions of varying sizes

**Figure 8:**
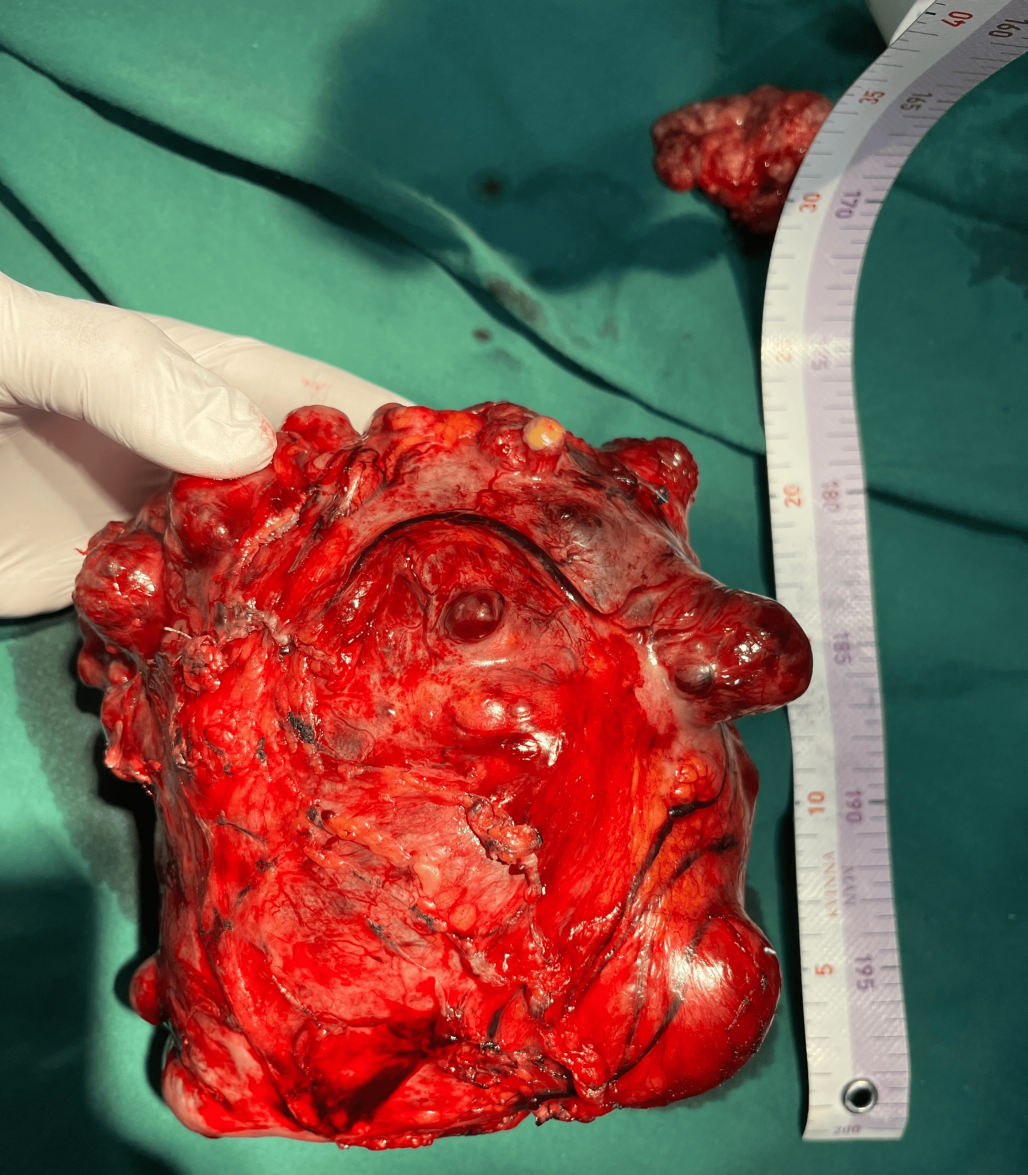
Specimen with numerous newly formed pathological blood vessels

**Figure 9:**
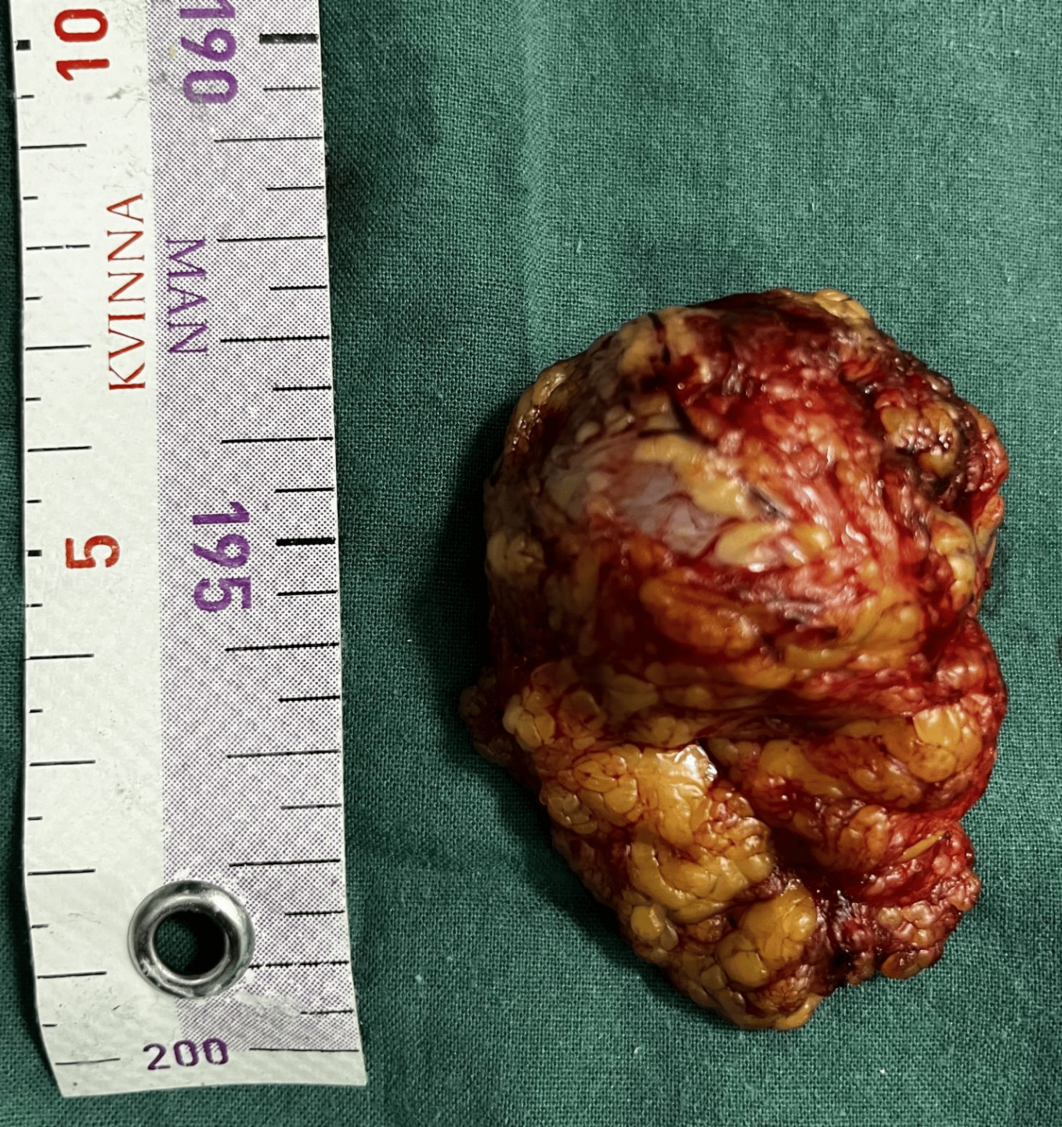
Metastasis in the larger omentum, 8 cm in length

During the laparotomy, we observed a well-defined, clearly circumscribed, irregular spherical tumor with many diverticular expansions of different sizes, many newly formed pathological blood vessels, a partially necrotic mass, and a partially hemorrhagic mass 20 x 20 cm in diameter. The tumor has adhered with dense adhesions in adjacent structures and organs (Figures 3, 4, 6-8). We performed an oncological (R0) resection along with a partial duodenum's D4 resection, which is the tumor's origin, and used interrupted sutures to stitch the duodenum back together (Figure 5). In the larger omentum, we found two metastases, each measuring approximately 8 x 5 cm (Figure 9). We were able to completely remove the tumor from its surrounding structures, only adhering to them and not invading. The postoperative course proceeded without complications, leading to the patient's discharge on the fifth postoperative day. After surgery, histopathology revealed that the patient had a GIST Grade II (high grade), high risk, pT4, Nx, M1, CD 117 positive, DOG1 positive, and KI 67.3%. The oncology council reviewed the case and recommended adjuvant treatment with tyrosine kinase-inhibiting agent imatinib mesylate (Gleevec).​​​​

## Discussion

Fifty to seventy percent of GISTs occur in the stomach (70% in the gastric body, 15% in the antrum, and 15% in the cardia) or small intestine (30% in the jejunum or ileum and 5% in the duodenum) [2]. Macroscopically, GISTs are white, well-defined, not encapsulated, and firm in consistency. Microscopically, the cellularity of GISTs can range from moderate to high, and three main types represent them: Spindle cell type (70%), epithelioid type (20%), and mixed type (10%) [2].

The pathological diagnosis of GIST is based on morphology and immunohistochemistry, which is typically positive for CD117 (KIT) and/or DOG1. About 80% of GISTs have changes in either the c-KIT or platelet-derived growth factor (PDGF) receptor [6].

The literature rarely describes cystic changes in GIST tumors, but a case report by Okano et al. and Gurram et al. shows that these changes are more common in malignant, high-grade GISTs [7,8]. Kawanowa et al. say that small GISTs happen in the stomach a lot more often than their estimated clinical prevalence suggests. This means that the frequency of GIST tumors in other places is probably a lot higher than we think [9]. When determining if a patient has GIST, one should consider four types of cancerous small bowel tumors: carcinoids (44.3%), adenocarcinomas (32.6%), lymphomas (14.7%), and leiomyosarcomas (1.2%) [10].

Due to the tumor's large mass and volume, it may have an impact on adjacent structures, potentially causing difficulties. Given the wide variety of illnesses that might manifest as retroperitoneal mass, additional diagnostic examinations are required. CT scans, MRIs, and ultrasounds can provide useful information on the mass's location, size, and features. Fluorodeoxyglucose positron emission computed tomography (FDG PET-CT) scanning is a highly sensitive method for detecting GIST and determining whether the tumor is viable [11].

The surgical management of duodenal cancers is challenging due to the complexity of the duodenopancreatic area. Because local lymph node involvement is uncommon in GISTs, the primary objective is to accomplish complete tumor resection with clear margins (R0 resection) without lymph node dissection. Surgical alternatives include local resection and pancreaticoduodenectomy, with equivalent disease-free survival rates for both procedures [12]. As in our case, segmental or wedge resection is an option for smaller lesions situated in the third or fourth duodenal portion, which is linked to a reduced incidence of complications. Pancreaticoduodenectomy is the primary treatment for extensive GISTs that infiltrate adjacent structures or involve the ampulla of Vater. Due to the localized growth's sole adherence to adjacent tissues, wedge resection was feasible, prompting the authors to opt for a thorough en-bloc surgery [13].

Despite complete surgical excision, GISTs frequently recur. Ng et al. found that only 10% of patients remained disease-free during long-term follow-up, but DeMatteo et al. discovered a 40% recurrence rate after two years [14,15]. Nearly two-thirds of recurrences showed hepatic involvement, with the liver being the only site in half of those cases. Recurrences might occur locally or in the peritoneum. Most recurrences occur during the first two years; however, low-grade tumors might resurface up to 10 years later. The management of recurrent illness can be challenging, with repeat curative resection potentially providing a minor survival benefit over palliative chemotherapy [14]. The risk of metastasis increases with tumor size, particularly when it exceeds 10 cm [15].

The survival rate of patients with GIST is contingent upon the following factors: the risk category or GIST stage, the treatment administered, and the likelihood of recurrence following treatment. As a result, individuals with localized GISTs have a five-year life expectancy of 93%, whereas those with locally progressed GISTs have a five-year survival rate of 80%, and patients with metastatic GISTs have a five-year survival rate of 55% [16].

Non-gastric GISTs had a lower disease-free survival rate compared to gastric GISTs. However, overall survival (OS) had no significant differences between the two groups. In this series, tumor size, location, and mean mitotic rates were associated with high recurrence rates as expected. In addition to these risk factors, high Ki67 levels were found to be associated with recurrence [17].

In their research, Sutton et al. demonstrated that patients with mGIST can achieve 10-year survivorship with the use of modern targeted therapies. The development of new therapies will only make this benchmark more achievable [18].

While surgery is the preferred treatment for the resectable disease, neoadjuvant therapy is recommended for advanced, metastatic, and recurrent tumors. A reduced tumor burden may enhance resection and diminish surgical morbidity. Antunes et al. exhibited a pathological complete response (pCR) with no residual cancer in their investigation. A complete histological response to imatinib in recurrent disease is exceptionally uncommon. In order to identify mutations that predict response, molecular testing should be conducted prior to neoadjuvant therapy [19].

OS is significantly longer in the surgery plus imatinib group. Some long-term analysis indicates that a significant number of patients experienced disease progression after discontinuing two-year maintenance imatinib therapy following surgery. Research on prolonged treatment durations in GIST patients at intermediate to high risk should be considered [20].

## Conclusions

GIST is a rare form of tumor; therefore, clinical evidence is insufficient to address numerous clinical and background questions. The standard clinical presentation, especially in young individuals with no familial history of gastrointestinal cancer, resembles prevalent gastrointestinal disorders, underscoring the necessity for precise diagnosis. Treatment modalities for symptomatic GIST encompass pharmacological or surgical intervention, contingent upon the tumor's dimensions, resectability, potential for malignancy, and the degree of metastatic involvement (if present). The successful surgical excision of the tumor and the metastasis is still the cornerstone of treatment for GIST. Given that molecular testing is mandated prior to neoadjuvant therapy to identify mutations that forecast response, we believe this case report is valuable as it demonstrates that complete R0 resection is frequently achievable, even in cases that appear inoperable, notwithstanding the surgical risks involved.
